# Elevated mortality among the second-generation (children of migrants) in Europe: what is going wrong? A review

**DOI:** 10.1093/bmb/ldad027

**Published:** 2023-11-01

**Authors:** Matthew Wallace, Lucinda Hiam, Robert Aldridge

**Affiliations:** Sociology Department, Stockholm University, Frescativägen, Stockholm 114 19, Sweden; School of Geography and the Environment, Oxford University Centre for the Environment, University of Oxford, South Parks Road, Oxford, OX1 3QY, UK; Institute of Health Informatics, University College London, 222 Euston Road London, NW1 2DA, UK; The Institute for Health Metrics and Evaluation, Hans Rosling Center for Population Health, 3980 15th Ave NE, Seattle WA 98195, United States

**Keywords:** mortality, second-generation, the children of migrants, inequality, public health, Europe

## Abstract

**Introduction:**

The ‘second-generation’ (i.e. the children of migrants) represent one of the fastest growing subpopulations of the child and young adult populations in Europe today. The research so far appears to indicate that their mortality risk is elevated relative to people with non-migrant backgrounds.

**Sources of data:**

Peer-reviewed publications.

**Areas of agreement:**

Second-generation status is a clear marker of elevated mortality risk in Europe in early life (including stillbirth, perinatal, neonatal and infant mortality) and adulthood, particularly if the parent(s) were born outside of Europe. Socioeconomic inequality plays an important, albeit rarely defining, role in these elevated risks.

**Areas of controversy:**

It remains unclear what causes-of-death are driving these elevated mortality risks. The exact influence of (non-socioeconomic) explanatory factors (e.g. health care, racism & discrimination, and factors related to integration) on the elevated mortality risks of the second-generation also remains unclear.

**Growing points:**

The second-generation will continue to grow and diversify in Europe; we must intervene to address these inequalities now.

**Areas timely for developing research:**

Place more emphasis on the complexity of migration background, specific causes-of-death, and understanding the roles of explanatory factors *beyond* socioeconomic background.

## Introduction

International migration has exerted salient demographic change upon the world in the past century; this is something that is likely to continue. This change is so great that the children of migrants (the so-called ‘second-generation’) comprise some of the fastest growing subpopulations of the infant, child and young adult populations across the world today.^1^ This includes Europe, where 31% of the world’s immigrants currently reside.^2^ This lasting demographic transformation has profound implications for population health, public health policy, clinical medicine, social policy, and research. While the evidence reveals that international migrants (the ‘first-generation’) have lower mortality rates than the non-migrant population of the host country (a ‘migrant mortality advantage’) that can last up to several decades after arriving in the host country,^3^^,^^4^ the evidence relating to their children (the ‘second-generation’) tells an altogether different story.

In this article, we synthesize the evidence relating to the mortality of the second-generation in Europe. All-cause mortality represents the ultimate health ‘endpoint’ and acts as a widely used summary measure of population health. Variation in all-cause mortality rates provides insight into how the overall levels of health of different subpopulations can vary.^5^ Differences in cause-specific mortality rates additionally offer insight into variation in the progression and severity of specific diseases and their associated risk behaviours.^5^ The review is structured as follows: we first outline how to define the second-generation (*Who are the Second-Generation?*) and offer a statistical overview of the second-generation in Europe today (*A Statistical Portrait of the Second-Generation*). We briefly describe our methods (*Methods*). Then, we summarize the evidence relating to the mortality of the second-generation in early life (*Early Life Mortality among the Second-Generation*) and adulthood (*Adult Mortality among the Second-Generation*) and outline the factors associated with their mortality. We close by recommending potential areas of intervention and future avenues of research (*Discussion*).

## Who are the second-generation?

The children of migrants—specifically children born in the country that their migrant parent(s) are residing in (the ‘host’ country)—are known as the ‘second-generation’. They can also be referred to as the ‘descendants’ or ‘offspring’ of migrants. Exactly how the second-generation are defined varies within and between countries, rendering comparisons of the evidence base difficult. This heterogeneity reflects a lack of universally accepted definition for ‘migrant’—including in international law. Anyone that moves from their usual of place residence, within a country or beyond international borders, for any amount of time, and for any reason can be considered a migrant.^6^ This lack of international consensus is complicated further by the use of different measures to determine migrant status that include nativity, nationality, citizenship and/or ethnicity.^7^ For this review, we consider migrants to be people living in a country other than their country of birth, as identified directly through their country of birth or indirectly by their citizenship or nationality. We then consider an individual born in a host country to *at least one* parent that meets these criteria to be second-generation.

## A statistical portrait of the second-generation

**Fig. 1:**
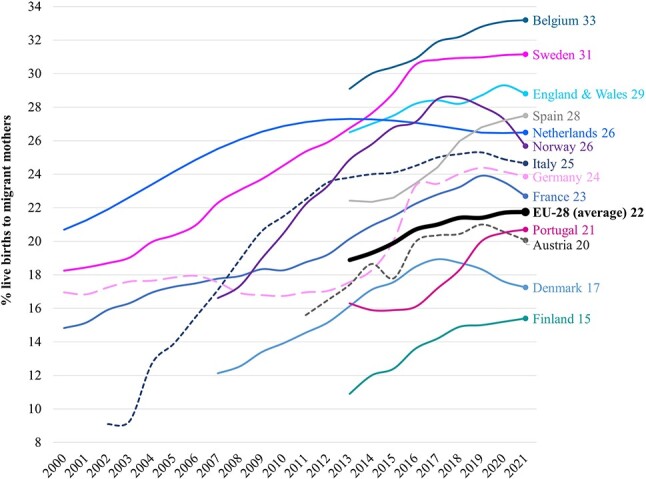
Live births to migrant mothers in selected European countries as a percentage of all live births in that country. *Notes*: (i) solid lines (the mother’s country of birth is used to define the second-generation), short dashed lines (the mother’s citizenship is used), long dashed lines (the mother’s nationality is used) (ii) the values next to the country names refer to the percentage of live births to migrant mothers in 2021. *Source*: Austria (Statistik Austria); Denmark (Danmarks Statistik); England and Wales (Office for National Statistics); France (Institut national de la statistique et des études économiques); Germany (Statistisches Bundesamt); Italy (I.Stat); the Netherlands (Caribisch Nederland); Norway (Statistisk sentralbyrå); Sweden (Statistiska centralbyrån). All other countries have been derived from Eurostat table ‘Live births by mother’s age and country of birth’ [Online data code: DEMO_FACBC]. Modifications to the Eurostat data have been made by the authors.

Second-generation populations in Europe are growing in size and becoming more and more diverse. The past several decades have witnessed the numbers of second-generation grow in absolute and relative terms in the context of the total resident populations of nearly all European countries. The relative growth over time in second-generation births is shown for selected countries in Figure 1. By 2021, 22% of all live births in the European Union (EU)/European Economic Area were to a migrant mother (Fig. 1). These relative numbers were even greater in major migrant-receiving countries that include Belgium (33%), England and Wales (29%), Germany (24%), Norway (26%) and Sweden (31%).

Table 1 then breaks down the number of second-generation births in 2019 (*the latest year for which data are available*) according to whether the migrant mother was born within or outside of the EU (for the same countries as Fig. 1). The majority of second-generation births in the EU were to a migrant mother born outside of the EU (74%; Fig. 1). Respectively, for Spain, Portugal, Sweden and France, these relative numbers are especially high at 82, 83, 85 and 88%.

**Table 1:** Second-generation births in 2019 by maternal birth region (EU-28 versus Non-EU)

Migrant mother born in:	EU-28	Non-EU	Total
France	12%	88%	100%
Sweden	15%	85%	100%
Portugal	17%	83%	100%
Spain	18%	82%	100%
Italy	22%	78%	100%
Finland	23%	77%	100%
**EU 28**	**26%**	**74%**	**100%**
Netherlands	27%	73%	100%
Germany	28%	72%	100%
Denmark	31%	69%	100%
Belgium	33%	67%	100%
Norway	36%	64%	100%
England and Wales	38%	62%	100%
Austria	41%	59%	100%

**Note:* the countries are ranked from top (highest) to bottom (lowest) according to the percentage of births to migrant mothers born outside of the EU-28. Source*: Derived from Eurostat table ‘Live births by mother’s age and country of birth’ [Online data code: DEMO_FACBC]. Modifications to the Eurostat data have been made by the authors.

In comparison, the relative numbers of adult second-generation are considerably lower and less diverse. Table 2 shows the adult second-generation as a percentage of total resident populations in 2021 (for the same countries as Fig. 1 and Table 1). The second-generation made up 7% of the total EU resident population aged 15–74, ranging from just 2.3% in Finland to 12.2% in Sweden. In all countries, the adult second-generation as a percentage of the total resident population aged 15-74 is largest in late adolescence and young adulthood. These numbers are especially high (compared with the EU average of 11%) in Austria (20.2%), Belgium (19.9%), the Netherlands (17%) and Sweden (18.5%).

**Table 2:** Percentage of the second-generation of total resident populations aged 15–74 in 2021

Countries	Age bands			
	15–74	15–29	30–54	55–74
Sweden	12.2	18.5	11.1	12.2
Belgium	12.1	19.9	12.0	12.1
Germany	11.9	16.6	9.0	11.9
France	11.7	12.8	13.5	11.7
Netherlands	10.7	17.7	10.0	10.7
Austria	10.5	20.2	7.3	10.5
England and Wales^†^	9.2	10.6	8.9	7.9
**EU 27**	**6.9**	**10.9**	**5.7**	**6.9**
Norway	6.1	12.6	4.7	6.1
Portugal	4.8	15.3	3.0	4.8
Denmark	3.4	6.2	2.4	3.4
Spain	2.8	8.9	1.6	2.8
Italy	2.6	9.2	1.2	2.6
Finland	2.3	6.8	1.2	2.3

*Source*: Derived from Labour Force Survey (LFS, 2021) online data browser table ‘population by the educational attainment level of the parents, sex, age, migration status and educational attainment level)’ [Online data code: LFSO_21EDUC08]. Modifications to the Eurostat data have been made by the authors.

^†^England and Wales derived from Labour Force Survey (LFS, 2014) online data browser table ‘population by age, sex, migration status, country of birth and country of birth of parents’ [Online data code: LFSO_14PCOB_P__ custom_7307880] (the last available data before the UK’s exit from the EU).

An earlier report (based upon the 2014 edition of the *Labour Force Survey* (LFS) from *Eurostat*) highlights that most of the adult second-generation in the EU aged 15–64 had migrant parent(s) born in other EU countries (72%).^8^ These percentages were larger in Finland (89%), Portugal (84%), Spain (85%) and Sweden (86%), and lower in Germany (60%) and the UK (67%).

Together, the patterns and trends described above reflect the earlier onset of intra-European migration (resulting in a largely *European-origin* adult second-generation) followed by more recent migrant inflows from outside of Europe (resulting in a predominantly *non-European-origin* infant second-generation).^9^

## Methods

In February 2023, searches were conducted in *Scopus*, *PubMed* and *Web of Science*. The search strings included a number of terms related to the second-generation (see ‘*Who are the second-generation*?’) and variations on terms related to mortality (e.g. survival, death, longevity) and its various stages (e.g. stillbirth, infant, neonatal, perinatal, adult). Searches were limited to the English language. Search strings are provided in Supplementary File S1. An update to this search was conducted in September 2023. As per the guidelines, we limited our reference list by prioritizing the inclusion of systematic reviews and of work post-dating the reviews. In total, we include three systematic reviews on mortality in early life,^10–12^ a ‘systematic review of systematic reviews’ on mortality in early life,^13^ and 22 studies post-dating the systematic reviews.^14–35^ For adult mortality, we include 21 studies.^1^^,^^36–53^

Supplementary File S2 provides a summary of the studies in relation to the outcome examined (perinatal, stillbirth, neonatal, infant, under-5-years old mortality and adult mortality), causes-of-death, the host country the study was conducted in and the origins of the migrant parent(s) of the second-generation (as categorized in the papers). Supplementary File S3 (*early life*) and Supplementary File S4 (*adult* mortality) additionally provide characteristics of each separate study. They outline the type of data used for the analysis, the precise definition of the second-generation (including the variables used) and the age ranges and time periods covered in each study.

From Supplementary Files S2–S4, we see that most of what we know about mortality among the second-generation in Europe originates from Belgium, Denmark, the Netherlands, Norway and Sweden. This reflects a greater availability of register data for research purposes in these countries and/or the longstanding status of the countries as major migrant host countries in Europe.^9^ A register is a digital collection of data that is collected and maintained for administrative purposes on people for the total population of a country. Information from these registers, which is collected and maintained by different government agencies or organisations (e.g. tax agencies, health agencies and welfare agencies), can be linked via a unique identity number. Register data is one of the few data sources that can meet the *extensive* demands that analyzing mortality patterns among the second-generation places upon the data. This can include a need for linkages between parents and children (to identify second-generation status), linkages to individual vital status, and linkages to registers that contain information on individual background characteristics (e.g., demographic, socioeconomic and geographic) that might conceivably explain differences in the mortality risks of the second-generation compared to non-migrants. However, as supplementary Files S3 and S4 also show, analyzing mortality among the second-generation is possible through the linkage of large-scale survey data to vital records.

We also see that the age ranges studied in early life mortality are standardized. This reflects the existence of legally defined and internationally agreed definitions of perinatal, stillbirth, neonatal and infant mortality. For *adult* mortality, we specify a definition of mortality from 15 years and older to account for variation in the age ranges. Some studies specify ‘open-ended’ intervals (e.g. 15+,^36^ 17+,^54^ 18+,^38^ 20+^53^); others limit their analyses to fixed intervals (e.g. 16–42,^55^ 15–44,^1^ 25–54,^41^ 18–64^50^^,^^51^). We did not find any studies of ‘old age’ mortality (i.e. from 65 years old), reflecting that the second-generation have yet to reach these ages in many European countries.

Finally, we see that in early life mortality the second-generation are always defined according to mother’s migrant status. This reflects the higher rates of missing paternal information for a pregnancy or birth,^14^ alongside the more salient role of biological, medical and demographic characteristics of the mother on the health of their child.^10^ For adult mortality, studies often adopt a wider definition, considering someone to be second-generation if they were born in the host country to *at least one* migrant parent. The increased relevance of the father’s migration status in second-generation adult mortality reflects a transition away from medical and health care-related factors (as explanations of mortality) and towards the social determinants of health (SDH).

*No new data were generated in support of this review.*

## Early life mortality among the second-generation

Table 3 summarizes the results from a series of meta-analyses that were conducted around 2009. Higher mortality is found among the second-generation for perinatal, stillbirth, neonatal *and* infant mortality. This disadvantage is most pronounced among second-generation with migrant mothers from non-European origins and those with migrant mothers who arrived as ‘refugees’^10^ (notably from former Yugoslavia; defined in the meta-analysis to include those who left home ‘unwillingly’ or had been in resettlement camps.). *Gagnon et al.*^11^ report higher early life mortality among second-generation with migrant mothers born in Asia, Northern Africa and Sub-Saharan Africa, but not among those with migrant mothers who were born in Latin America or other parts of Europe. This geographic variation *broadly* matches the description of mortality variation by mothers’ birth country in *Gissler et al*.^10^*and* the variation found in the studies postdating these reviews.^14–35^ Simultaneously, these estimates mask a large amount of heterogeneity that reflects the sheer diversity of the second-generation in Europe today. Looking within the world regions used to categorize mother’s birth country in Table 3, we see marked variation in early life mortality risk. Second-generation born to migrant mothers born in Asia are a prime example. Five studies find elevated stillbirth and infant mortality in second-generation born in Denmark and Norway to migrant mothers born in Sri Lanka and Pakistan, but not Vietnam or Thailand.^19^^,^^23^^,^^26^^,^^27^^,^^32^ Another study documents elevated neonatal and infant mortality among second-generation born in England and Wales to migrant mothers born in Bangladesh and Pakistan, but not in India.^30^ There are also cases where early life mortality among the second-generation with migrant mothers born in the same birth country/region varies by the mother’s host country. For example, second-generation with migrant mothers born in Northern Africa have a high early life mortality risk if they were born in France^10^^,^^31^ and Belgium,^16–18^ but not if they were born in the Netherlands^10^ or Norway.^10^^,^^27^

**Table 3:** Summary of results from meta-analyses on early life second-generation mortality in Europe

Study	Outcome	G2 group	Metric	Est.	95% CIs		
Gissler *et al*. (2009)	Stillbirth	All G2	RR	1.40	1.22	–	1.58
	Perinatal		RR	1.35	1.26	–	1.45
	Neonatal		RR	1.34	1.30	–	1.38
	Infant		RR	1.33	1.30	–	1.36
	Stillbirth	Non-Western	RR	1.88	1.58	–	2.23
	Perinatal		RR	1.54	1.39	–	1.69
	Neonatal		RR	1.40	1.36	–	1.44
	Infant		RR	1.37	1.34	–	1.40
	Stillbirth	Refugee	RR	2.01	1.47	–	2.73
	Early neonatal (i)		RR	2.77	1.85	–	4.13
	Perinatal		RR	1.71	1.41	–	2.06
Gagnon *et al*. (2009)	Feto-infant (ii)	Asia	aOR	1.29	1.02	–	1.63
		North Africa	aOR	1.25	1.10	–	1.41
		Intra-Europe	aOR	1.14	0.75	–	1.72
		Latin America	aOR	1.02	0.75	–	1.39
		Sub-Saharan Africa	aOR	2.43	0.99	–	5.96
Bollini *et al*. (2009)	Perinatal	All G2	OR	1.50	1.47	–	1.53
		All G2 in host countries with weaker integration policies	OR	1.56	1.52	–	1.60
			aOR	1.45	1.13	–	1.86
		All G2 in host countries with stronger integration policies	OR	1.41	1.37	–	1.46
			aOR	1.25	1.17	–	1.34

*Sources:* Estimates from pages 136 and 139 in Gissler *et al*. (2009) reprinted from Gissler M, Alexander S, Macfarlane A, et al. Stillbirths and infant deaths among migrants in industrialized countries. Acta Obstet Gynecol Scand 2009;88:134–48 with permission from John Wiley and Sons^10^; Estimates from Figure 1 in Gagnon *et al*. (2009) reprinted from Gagnon AJ, Zimbeck M, Zeitlin J, et al. Migration to western industrialised countries and perinatal health: a systematic review. Soc Sci Med 2009;69:934–46 with permission from Elsevier^11^; Estimates from Table 3 and Table 4 in Bollini *et al*. (2009) reprinted from Bollini P, Pampallona S, Wanner P, et al. Pregnancy outcome of migrant women and integration policy: a systematic review of the international literature. Soc Sci Med 2009;68:452–61 with permission from Elsevier.^12^

*Notes*: (i) early neonatal mortality (a death occurring 0–6 days after birth); (ii) feto-infant mortality (neonatal mortality, infant mortality and spontaneous abortions). (iii) adjusted odds ratios (aORs) in Bollini *et al*. (2009) additionally adjusted for age at delivery and parity; aORs in Gagnon et al. (2009) only stated to adjust for between four to eight (unnamed) covariates (iii) data from the 22 studies post-dating the reviews are not included in the meta-analyses reported for these systematic reviews.

**Fig. 2:**
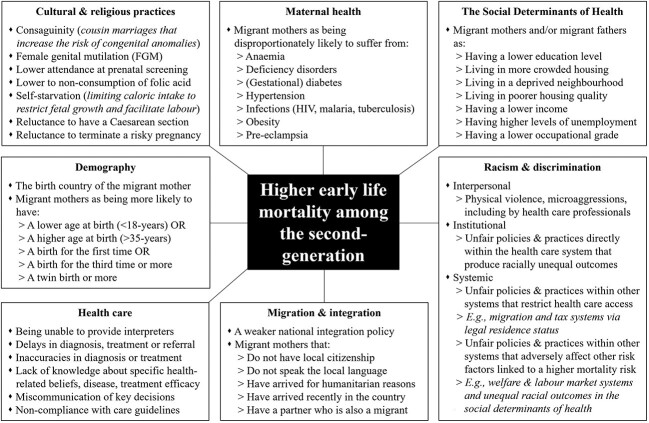
Factors most typically associated with higher early life mortality in the second-generation. *Notes*: (i) the list is non-exhaustive. It documents those factors most often ‘adjusted for’ in statistical analyses of early life mortality and/or factors discussed as potential explanations of the higher early life mortality of the second-generation in papers included in this review (ii) the boxes are placed in alphabetical order (from column-to-column), as are the specific factors within each of the boxes (from top-to-bottom).

Nevertheless, certain groups do face persistent excess early life mortality risks in Europe today. Of all the second-generation defined in the studies, those with migrant mothers born in Turkey,^10^^,^^16–18^^,^^20^^,^^22^^,^^23^^,^^27^^,^^32^^,^^33^^,^^35^ Sub-Saharan Africa^10^^,^^15^^–^^19^^,^^22^^,^^23^^,^^25^^,^^27–32^^,^^35^ (notably Somali^10^^,^^19^^,^^22^^,^^23^^,^^27^^,^^32^^,^^35^) and Pakistan^10^^,^^19^^,^^22^^,^^23^^,^^26^^,^^27^^,^^30^^,^^32^^,^^35^ are consistently exposed to a higher risk of death in early life within Europe. This holds true before *and* after adjusting for a range of potential factors that might help to ‘explain’ their increased early life mortality risk as compared to children born to non-migrants (see Fig. 2).

Beyond mortality from all causes of death, congenital anomalies (particularly among second-generation with migrant mothers born in Sub-Saharan Africa, the Middle East and North Africa),^10^^,^^15^^,^^17^^,^^18^^,^^22^^,^^35^ hereditary metabolic diseases,^10^ infectious diseases,^10^^,^^15^ prematurity conditions,^18^ intrapartum events (including birth asphyxia),^18^ and accidents^10^^,^^35^ have been identified as *potentially* relevant causes-of-death. However, the evidence relating to the role of specific causes-of-death in variation in all-cause early life mortality remains extremely limited.

Figure 2 provides a list of the demographic, health, migration and socioeconomic factors typically associated with a higher early life mortality risk among the second-generation. The factors are myriad, complex, interrelated, non-exhaustive and likely vary in their effect contingent on the birth country of the migrant mother and/or host country that they are living in. For example, *racism and discrimination* can directly affect the risk of early life mortality through acts of interpersonal violence. It may also indirectly affect early life mortality through the development of *maternal health* conditions (e.g. chronic stress) that increase the risk of prematurity or lower birthweight, and through racially unequal outcomes in *SDH* and *health care.*^56–58^ The extent of any racism is influenced by the strength of a host country’s *integration* policy and its effect on institutional, structural and personal attitudes towards migrants^12^ and, ultimately, the country of birth of the mother.

In general, studies find that these background factors can explain some, but not all, of the higher early life mortality risk of the second-generation—sizeable amounts of elevated mortality risk persist.^10^

## Adult mortality among the second-generation

Studies of all-cause mortality find evidence of higher adult mortality risks among second-generation with migrant parent(s) born outside of Europe relative to adults born in the host country to two parents born in the host country.^1^^,^^39^^,^^41^^,^^49–51^^,^^53^ Most of this higher mortality risk is concentrated among men^1^^,^^39^^,^^41^^,^^50^^,^^51^^,^^55^ and it presents most consistently among second-generation with migrant parent(s) born in the Middle East, North Africa (MENA)^1^^,^^39^^,^^41^^,^^49–51^^,^^55^ and Sub-Saharan Africa.^41^^,^^49^^,^^53^^,^^55^ In contrast, there is less evidence of higher mortality among the European-origin second-generation.^1^^,^^39^^,^^41^^,^^50^^,^^51^ There are even cases of lower mortality among second-generation adults with migrant parent(s) born in Southern Europe.^41^^,^^50^^,^^51^ However, there is also some evidence of higher mortality risks among second-generation with migrant parent(s) born in: Ireland living in England and Wales,^36^ France in Belgium,^41^ and Finland^1^^,^^39^^,^^55^ and former Yugoslavia living in Sweden.^39^^,^^55^

Something that sets the adult mortality literature apart from the early life mortality literature is the ability to produce mortality estimates for migrants and compare mortality across generations. This is all but impossible for early life mortality due to the lack of arrivals of very young children arriving from another country in the first year of life—and particularly in the first 28 days when risk of infant mortality is highest^59^. Doing so offers considerable insight into the adult mortality variation described above. Seven of the twelve all-cause mortality studies provide estimates for first-generation migrants.^1^^,^^36^^,^^41^^,^^49^^,^^51–53^ They show that when the mortality risk of second-generation with non-European origins is *higher* than the mortality risk of non-migrants in Europe, the mortality risk of their respective migrant groups is almost always *lower*. This is the case for first and second-generation with MENA,^41^^,^^49^^,^^51^ Sub-Saharan African,^41^^,^^49^^,^^53^ Asian,^1^^,^^53^ Central and Southern American,^1^ and Caribbean backgrounds.^53^ Conversely, in the limited cases where the mortality risk of second-generation with European origins is *higher,* mortality among the first-generation is higher as well.^1^^,^^36^^,^^41^^,^^53^ Crucially then, while the higher mortality of second-generation with European origins reflects the perpetuation of the initial higher risk experienced by a small number of first-generation migrants groups, the higher mortality risks of second-generation adults with non-European origins represent a systematic reversal from a position of relative mortality advantage to one of disadvantage between the first and second-generations. It is important to note, however, that these comparisons do not really compare parents with children but concurrent generations of migrants and the children of migrants.

Studies on cause-of-death in the second-generation in adulthood are scarce and paint a complex picture of specific risks according to the country of birth of their migrant parent(s) and the host country that they are living in. Some studies report increased mortality from *all external causes-of-death combined,* including adults with migrant parent(s) born in Finland, former Yugoslavia, MENA and Sub-Saharan Africa residing in Sweden^1^^,^^39^^,^^55^ and Ireland residing in England and Wales.^36^*Accident and injury mortality* is elevated among adults with migrant parent(s) born in Finland and Sub-Saharan Africa living in Sweden^1^ and Ireland living in England and Wales.^36^*Substance use mortality* is higher among adults with migrant parent(s) born in Northern Africa living in Belgium^51^ and in all second-generation groups in Sweden except Sub-Saharan Africa^1^^,^^38^—particularly high relative risks are reported among adults with migrant parent(s) born in Finland, Central and Eastern Europe and MENA countries.^1^ Studies on *suicide mortality* report a higher risk in second-generation with migrant parent(s) born in other Nordic countries living in Norway^45^ and Sweden.^1^^,^^46^^,^^48^ This contrasts with the lower mortality risk among adults with migrant parent(s) born in MENA residing in Belgium (see Morocco and Turkey)^47^ and Sweden.^1^^,^^46^

Regarding natural causes-of-death, the risk of *all cancer mortality* often closely resembles that of adults born to non-migrants, irrespective of the birth country of the migrant parent(s) or host country.^1^^,^^37^^,^^40–43^ Nevertheless, people with migrant parent(s) born in France and Morocco residing in Belgium stand out for their high relative *all cancer mortality* risks.^41^^,^^43^ For *lung and liver cancer*—which more closely reflect lifestyle factors such as diet, smoking and drinking—studies report raised mortality among adults with migrant parent(s) born in France and Morocco living in Belgium,^41^^,^^43^ Ireland in England and Wales,^36^ and Indonesia in the Netherlands,^37^ but lower mortality in second-generation with other Western-European (non-French) origins living in Belgium.^41^^,^^43^ In Sweden, *alcohol-related mortality* is lower for adults with migrant parent(s) born in non-Nordic countries living in Sweden^38^—compared with adults born to non-migrants—but higher among adults with migrant parent(s) born in Finland.^54^*Circulatory disease* (and notably *coronary heart disease*) mortality is elevated among adults with migrant parent(s) born in Turkey living in Belgium and Sweden,^41^^,^^44^ France and Morocco in Belgium,^41^ and Central and Eastern Europe, Denmark, Finland, and Norway in Sweden.^1^^,^^44^*Infectious disease mortality* (including HIV and Hepatitis) is elevated among adults with migrant parent(s) born in France, Morocco, Turkey and Sub-Saharan Africa living in Belgium^41^ and Indonesia in the Netherlands.^37^

Importantly, the prevalent role of external causes among certain second-generation groups is a function of their young age profiles. Many second-generation groups (notably subgroups with non-European origins) are concentrated at ages around the ‘mortality accident hump’, an age range between 15 and 40 in which mortality is driven by causes like accidents and injuries, suicides, homicides and substance use. As such, any differences in all-cause mortality around these ages will almost always be attributable to observed differences in external causes-of-death. On the other hand, the more prevalent role of natural causes in European-origin groups with a higher mortality risk reflects that the groups are older and have reached ages where diseases and medical conditions have begun to play a more defining role in all-cause mortality risk.

**Fig. 3:**
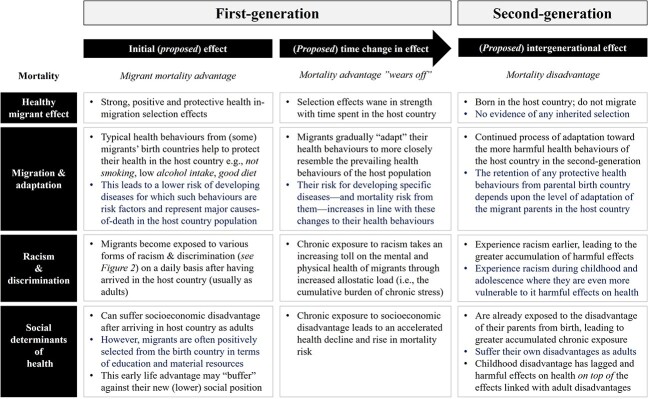
How factors affecting mortality risk might differ between adult first- and second-generation.

Many of the background factors relevant to early life mortality in Figure 2 remain relevant to the adult mortality of the second-generation (e.g. factors related to *health care*, *migration and integration*, *racism and discrimination* and *the SDH*). With this in mind, Figure 3 offers an intergenerational perspective that outlines how researchers currently theorize how some of these factors might differ between the first and second-generations to produce the divergent adult mortality risks of (particularly non-European) migrants and their children that we see in Europe today.

Importantly, the strength and (even the direction) of the effect of these factors on mortality from Figure 3 can vary according to the birth country of migrant parent(s). For example, the *healthy migrant effect* is typically conceptualized through a lens of positive health selection. Yet, some migrant groups are negatively selected upon factors linked to poor health (e.g. low education, blue-collar work and unemployment). In these cases, their parent(s) arrive with a high mortality risk that perpetuates in the mortality risk of their children. The same is true for migration, adaptation, and health behaviors. While it is assumed that the loss of health behaviors associated with migrants’ birth countries will be detrimental to the health of migrants and their children, some migrant groups practice unhealthy behaviors associated with their birth country that adversely affect their mortality risks (e.g., higher levels of smoking and drinking). As such, the loss of such behaviors among their children would instead be beneficial. Both of these cases apply to Irish, French and Finnish migrants and their children living in England and Wales,^36^ Belgium,^41^ and Sweden^1^^,^^39^^,^^55^, respectively. We know that these groups are (at least historically) negatively selected upon many SDH and practice behaviors detrimental to their health.

## Discussion

### Summary

With the general exception of second-generation with European origins, who have comparable early life and adult mortality to individuals born in the host country to non-migrant parents, the evidence reveals higher early life and adult mortality risks among second-generation with non-European origins. Of all the second-generation groups, children with migrant mothers born in Turkey, Somalia and Pakistan face the most sizeable and persistent early life mortality risks. In adulthood, second-generation with migrant parent(s) born in MENA countries and Sub-Saharan Africa face higher all-cause mortality risks. Congenital anomalies, hereditary diseases, prematurity and intrapartum events are *potentially* important causes-of-death in early life. In adulthood, the evidence points to a differing impact of natural and external causes contingent upon the country of birth of the migrant parent(s) and the ages at which specific second-generation groups are concentrated. The evidence relating to adult causes-of-death is *very* limited. Yet, we must also take care not to over-generalize. Not all second-generation with migrant parent(s) born outside of Europe have high mortality compared with those born to non-migrants. Similarly, not all second-generation with migrant parent(s) born in other parts of Europe have similar risks to those born to non-migrants. Various examples are highlighted throughout the course of this review.

### The European situation in context

In *early life*, the mortality situation in Europe appears to be comparable with the mortality situation in North America. In Canada, second-generation infant mortality levels are considerably lower *overall*, but different subpopulations encounter different risks similar to the mortality patterns described in Europe. This includes heightened risks among children with mothers born in the Caribbean, Haiti, Pakistan and Sub-Saharan Africa (i.e. non-Western), alongside reduced risks in children with mothers born in the USA and Europe (i.e. Western).^60^ In the USA, second-generation infant mortality is higher *overall*. Yet, some groups encounter an elevated infant mortality risk relative to the US-born non-Hispanic White population (children of mothers born in the Pacific Islands, Puerto Rico and Sub-Saharan Africa), while others encounter a lower risk (children of mothers born in Asia [China, Japan, the Philippines, India, Korea], Cuba and Central Southern America).^61–63^

It is in *adulthood* that the European and US situations diverge. Although adult mortality rates among the second-generation in the USA are not as low as they are amongst the first-generation, they tend to remain lower than the mortality rates of US-born non-Hispanic Whites (i.e., the non-migrant population). US-born Hispanics (particularly Mexicans and Cubans) and Asian and Pacific Islanders aged 25–64 and 65+—two of the larger subpopulations of second-generation in the USA—retain the lower all-cause, cancer, cardiovascular and respiratory mortality rates of first-generation migrants.^63^^,^^64^ Ultimately, this means that the second-generation have helped to elevate national life expectancy levels in the USA in recent decades.^65^ The same cannot be said in Europe—or at the very least in Sweden—where the second-generation (all parental origins) have been contributing increasingly negatively to national life expectancy levels in the past 30 years.

### Recommendations for public policy and practice

The heterogeneity in mortality risk according to the birth country of the migrant parent(s) and the host country reflects the complex interplay—and varying effect—of the background factors combining to generate the mortality risks of the second-generation. In early life and adulthood, socioeconomic disadvantage often plays an important role as an explanatory factor between the second-generation and those born to non-migrants, even if some of the higher mortality risks of the second-generation remain. It is true, however, that measures of socioeconomic disadvantage (e.g. education level, income and unemployment) are the type of background factors most readily available in data sources used to examine mortality among the second-generation. Many of the background factors that we theorize to be important (e.g. racism and discrimination, integration and in-migration selection effects) are difficult to conceptualize, let alone adequately measure and test. Even information on more ‘tangible’ factors, such as the health status of the mother (in early life mortality), is not routinely linked to mortality data. Consequently, it is rarely possible to perform analyses of mortality among the second-generation that afford equal consideration to all relevant background factors.

This renders the design of effective policies and interventions difficult. Nevertheless, three common areas of intervention could help us to ameliorate the mortality situation of all second-generation.

First, the clear division in mortality situation of the second-generation according to whether or not their migrant parent(s) were born within or outside of Europe further emphasizes the urgent priority to tackle racism, xenophobia and discrimination within wider society and the European health care systems. They represent fundamental determinants of health and must be considered as such when formulating approaches to public health. This focus will help to address known causes of mortality in early life such as late and poor access to antenatal care, but would also begin to tackle structural issues that are likely to underline higher adult mortality from external causes.

Second, it is essential to re-evaluate the effectiveness of national migrant integration policies. The second-generation as having comparable life outcomes with those born in the host country to two non-migrant parents is regarded as one of the key markers of the successful integration of migrant populations.^1^ That the second-generation in Europe are exposed to an elevated risk of mortality from birth right through to adulthood is alarming and suggests that much more could be done to protect migrants and their children and promote their economic integration and fuller participation in wider society. Future migration integration policies must as a matter of urgency acknowledge and explicitly engage with the second-generation issues highlighted here and take a comprehensive intergenerational approach to migration and health that has been lacking in policies so far.

Third, SDH are regularly found to play an important role in explaining the higher mortality of the second-generation in the studies incorporated in this review. As such, continued action towards reducing inequalities in SDH, like education, income, social protection, working life conditions, unemployment and job insecurity, can only have a positive effect upon the early life and adult life chances of the second-generation within Europe.

### Recommendations for future research

To improve our understanding of the mortality patterns of the second-generation and to better inform policies aimed at improving their mortality situation, Box 1 proposes futures avenues of research.

**Box 1:** Proposed research agenda for second-generation mortality in Europe

**Establish whether the situation is getting better or worse.**
*How are absolute and relative mortality risks among the second-generation changing over time? Are differences to the non-migrant population increasing, decreasing, or remaining stable?*
**Understand if the patterns represent all of Europe.**
*Most of the evidence covered here comes from a few countries because data are more readily available in those countries. In countries where register data exist (but are not easily available for research purposes) it could be made more readily available. In the countries that rely more upon census and surveys linked to mortality data, one could begin to ask questions on parental birth country as a standard to permit analyses of mortality among the second-generation.*
**Identify the main causes-of-death.**
*Move beyond all-cause mortality and towards specific causes-of-death that can provide a more focused insight into the explanations behind the high mortality of the second-generation (e.g. health and risk behaviours) and more targeted evidence for new public health policies.*
**Embrace complexity in migrant background.**
*Define the origins of the second-generation to the lowest level parental country of birth possible to avoid masking potential inequalities in specific groups. Incorporate other important migrantbackground factors, such as the reason for arrival (e.g., work, family reunification, refugee) and the number of migrant parents (i.e., one versus two) in order to generate new insight into explanatory mechanisms like integration and adaptation*
**Test other mechanisms.**
*Many of the studies account for the Social Determinants of Health because this type of information is more readily available. Yet, a growing number of surveys ask about e.g. perceived racism and discrimination and/or experiences with health care. Can these surveys be linked to longitudinal data on mortality in the second-generation and tested as explanatory mechanisms?*

## Conclusion

Second-generation status is clearly a marker of elevated mortality risk in Europe. Although absolute mortality risks in infancy (320 deaths per 100,000 live births in 2021)^66^ and particularly young adulthood (ages 15-29, 36 deaths per 100,000 residents in 2018)^67^ in Europe (or more specifically the European Union) are low and have been falling steadily over time, the increased early life and adult mortality risks of the second-generation should not be ignored. A premature death constitutes the most fundamental of all of life’s inequalities; every other type of inequality is contingent upon being alive.^1^ People who migrate to a new country do so with hopes for a better future for themselves and their children. This hope—at least with respect to expectations of life—has so far failed to materialize in Europe. The second-generation are losing decades of potential life via avoidable mortality from causes that are preventable (i.e. via effective social, public health and primary interventions) and/or treatable (i.e. via timely and effective health care intervention [secondary prevention and treatment]). The people behind the numbers should not be forgotten. Something is going wrong in Europe; it should be a priority to find out what.

## Glossary

### Adult mortality

Death on or after age 15 years old (*World Health Organisation*).

### Birth country

The country in which the migrant parent(s) of the second-generation were born in (and migrated from).

### External causes-of-death

Mortality due to accidents and violence that can include environmental events, circumstances and conditions as the cause of injury, poisoning and other adverse implications (*World Health Organisation*).

### Host country

The country in which the migrant parent(s) are living in (and the second-generation were born in).

### Infant mortality

Death within the first 365 days of life (*World Health Organisation*).

### Integration

The process by which migrants become accepted into society, both as individuals and as groups. Integration refers to a two-way process of adaptation by migrants and host societies. It implies the consideration of the rights and obligations of migrants and host societies, access to different kinds of services and the labour market, and of identification and respect for a core set of values that bind migrants and host communities in a common purpose (*International Organisation for Migration*).

### Migrant

An umbrella term, not defined under international law, for a person who moves away from his or her place of usual residence, within a country or across an international border, temporarily or permanently, and for a variety of reasons (*International Organisation for Migration*). Here, we refer to migrants as individuals residing in a country other than their country of birth, as identified directly by their country of birth or indirectly through having foreign citizenship or nationality.

### Natural causes-of-death

Mortality resulting from diseases, medical conditions and/or natural processes (*World Health Organisation*).

### Neonatal mortality

Death within the first 28 days of life (*World Health Organisation*).

### Perinatal mortality

The number of foetal deaths past 22 (or 28) completed weeks of pregnancy plus the number of deaths among live-born children up to the first seven completed days of life (*World Health Organisation*).

### Second-generation

The children of migrants—specifically children born in the country that their migrant parent(s) are residing in (the ‘host’ country). In early life mortality studies (defined here as death before 15 years old), the second-generation are defined exclusively according to the migrant status of the mother. For adult mortality (defined here as death on or after 15 years old), studies tend to adopt a much more inclusive definition, considering an individual to be second-generation if they born in the host country to at least one migrant parent (i.e. a migrant mother and/or migrant father).

### Social determinants of health

Non-medical factors that affect health. They are the conditions in which people are born, grow, work, live and age, and the wider set of forces and systems shaping the conditions of daily life. These forces and systems include economic policies and systems, development agendas, social norms, social policies and political systems. Examples include income and social protection, education, unemployment and job insecurity, working life conditions, housing, basic amenities, early child development, social inclusion and non-discrimination (*World Health Organisation*).

### Stillbirth

A baby who dies after 28 weeks of pregnancy, but before or during their birth (*World Health Organisation*).

## Supplementary Material

Suppl_file_S1_ldad027

Suppl_file_S2_ldad027

Suppl_file_S3_ldad027

Suppl_file_S4_ldad027

Supplementary_materials_ldad027
