# Supplementary material for: Elevated mortality among the second-generation (children of migrants) in Europe: what is going wrong? A review
Source: Br Med Bull. 2023 Nov 1;148(1):5–21. doi: 10.1093/bmb/ldad027 (PMC10724460; doi:10.1093/bmb/ldad027)
Supplement: Suppl_file_S1_ldad027 [file suppl_file_s1_ldad027.pdf]

**Supplementary file S1.** Database searches

***Scopus***

( TITLE-ABS-KEY ( mortality OR death OR infant AND mortality OR perinatal AND mortality OR neonatal AND mortality OR infant AND death OR perinatal AND death OR neonatal AND death OR life AND expectancy OR longevity OR surviv* OR stillbirth ) AND TITLE-ABS-KEY ( second-generation OR child* OR descendant* OR offspring ) AND NOT TITLE-ABS-KEY ( cell* OR surgery OR molecul* OR clinic* OR antipsy* OR drug* OR treatment OR protein OR kidney OR vaccin* OR pollut* ) ) AND NOT TITLE-ABS-KEY ( "soil" OR "vaccin*" OR "*biolog*" OR "bird*" OR "cancer" OR "veteri*" OR "DNA" OR "genetic*" OR "gene" OR "genes" OR "cell*" OR "tox*" OR "medic*" OR "animal*" OR "disease*" ) AND NOT ( SUBJAREA ( agri OR bioc OR immu OR neur OR phar ) OR SUBJAREA ( ceng OR chem OR comp OR eart OR ener OR engi OR envi OR mate OR math OR phys ) )

***Web of Science***

((ALL=(mortality OR death OR perinatal mortality OR neonatal mortality OR infant death OR perinatal death OR neonatal death OR longevity OR surviv* OR stillbirth)) AND ALL=(second-generation OR descendants of immigrants OR descendants of migrants OR children of immigrants OR children of migrants OR offspring of immigrants OR offspring of migrants OR immigrant descendants OR migrant descendants OR immigrant children OR migrant children OR immigrant offspring OR migrant offspring))

***PubMed***

((mortality OR death OR infant mortality OR perinatal mortality OR neonatal mortality OR infant death OR perinatal death OR neonatal death OR life expectancy OR longevity OR surviv* OR stillbirth) AND (second-generation OR descendants of immigrants OR descendants or migrants OR children of immigrants OR children of migrants OR offspring of immigrants OR offspring of migrants OR immigrant descendants OR migrant descendants OR immigrant children OR migrant children OR immigrant offspring OR migrant offspring) AND ((excludepreprints[Filter]) AND (humans[Filter]) AND (english[Filter])) AND ((excludepreprints[Filter]) AND (humans[Filter]) AND (english[Filter])) AND ((excludepreprints[Filter]) AND (humans[Filter]) AND (english[Filter]))) NOT (randomized) NOT (drug*) NOT (trial) NOT (case report) NOT (clinic) NOT (antipsy*) NOT (surgery) AND ((excludepreprints[Filter]) AND (humans[Filter]) AND (english[Filter])) AND ((excludepreprints[Filter]) AND (humans[Filter]) AND (english[Filter])) AND ((excludepreprints[Filter]) AND (humans[Filter]) AND (english[Filter])) AND ((excludepreprints[Filter]) AND (humans[Filter]) AND (english[Filter])) AND ((excludepreprints[Filter]) AND (humans[Filter]) AND (english[Filter])) AND ((excludepreprints[Filter]) AND (humans[Filter]) AND (english[Filter])) AND ((excludepreprints[Filter]) AND (humans[Filter]) AND (english[Filter])) AND ((excludepreprints[Filter]) AND (humans[Filter]) AND (english[Filter])
