# Supplementary material for: Elevated mortality among the second-generation (children of migrants) in Europe: what is going wrong? A review
Source: Br Med Bull. 2023 Nov 1;148(1):5–21. doi: 10.1093/bmb/ldad027 (PMC10724460; doi:10.1093/bmb/ldad027)
Supplement: Suppl_file_S2_ldad027 [file suppl_file_s2_ldad027.pdf]

Supplementary file S2. Basic information on the studies incorporated into the review.

Outcome	Cause-of-death	Host country	Birth country/region of migrant parents
Perinatal<br>(5 studies; 2 systematic reviews**: 2 meta-analyses**)	Asphyxia and unexpected deaths prior to onset of labour [13], congenital malformations [10,12,13], infections [10], intrapartum events [13], prematurity and foetal growth [10,13]	Spain [10], Belgium [11–13], Norway [14]	**European**: All EU-27 [11], new EU-27 [12], other EU-15 [12], Western Europe [10], Eastern Europe [10–12]
			**Non-European:** Turkey [11–13], Maghreb region [11], Northern Africa [10,12], Morocco [13], Sub-Saharan Africa [10–13], Somalia [14], Afghanistan [14], Iraq [14], Asia [10], Pakistan [14], Philippines [14], Sri Lanka [14], Thailand [14], Vietnam [14], Latin America [10]
Stillbirth<br>(11 studies; 1 systematic review**: 1 meta-analysis**)		Austria [15], Belgium [15,16], Denmark [15,17,18], Germany [15,19], Netherlands [20], Norway [5,15,21,22], Spain [23], Sweden [15,24], Switzerland [15], United Kingdom [15]	**European**: EU [23], Western Europe [24], Germany [18], Iceland [18], Norway [18], Denmark [22], Sweden [18,22], Mediterranean countries [19], Eastern Europe [24], former Yugoslavia [17,18,22], other Europe [23], Poland [18,22], Romania [18], Russia [22], Ukraine [18], Lithuania [18],
			**Non-European:** Turkey [15,17,18,22], Turkey/Morocco [20], Africa [20,24], Morocco [22], Sub-Saharan Africa [23], Somalia [17,18,22], Middle East [24], Afghanistan [18,22], Iran [18,22], Iraq [18,22], Lebanon [17,18], Syria [18] Asia [20,24], East Asia [20], Asia & Oceania [23], Pakistan [17,18,21,22], Philippines [18,22], Sri Lanka [22], Thailand [18,22], Vietnam [18,22], China [18], Latin America [24], South America & the Caribbean [23]
			**Other:** All [5,16], high-income countries [16], middle income-countries [16], low-income countries [16], Europe & America [19], other Western [20], other non-Western [20]
Neonatal<br>(4 studies; 3 systematic reviews**; 1 meta-analysis**)		Belgium [16], Netherlands [20], Spain [10], United Kingdom [25]	**European**: Western Europe [10], other Western [20], Eastern Europe [10]
			**Non-European:** Turkey/Morocco [20], Africa [20], Northern Africa [10], Sub-Saharan Africa [10,25], Asia [10], East Asia [20], South Asia [20], India [25], Pakistan [25], Bangladesh [25], Latin America [10], Caribbean [25]
			**Other:** All [16], high-income countries [16], middle income-countries [16], low-income countries [16], White [25], other non-Western [20],
Infant<br>(9 studies; 2 systematic reviews**; 2 meta-analyses**)	Congenital malformations [17], , perinatal causes [17], Sudden Infant Death Syndrome [17]	Denmark [17,18], Belgium [16] France [26], Norway [21,27], Switzerland [28,29], United Kingdom [25]	**European**: Europe [27], Germany [18,27,28], Iceland [18], Norway [18], Sweden [18,27], Denmark [27], United Kingdom [27], France [28], Italy [28], Portugal [28], Spain [28], Eastern Europe [26], former Yugoslavia [17,18], Kosovo [28], Macedonia [28], Poland [18,26,27], Romania [18,26], Russia [26], Lithuania [18], Ukraine [18]
			**Non-European:** Turkey [17,18,26–28], Africa [27], Northern Africa [26], Algeria [26], Morocco [26], Tunisia [26], Sub-Saharan Africa [25,26], Western Africa [26], Cameroon [26], Comoros [26], Democratic Republic of Congo [26], Guinea [26], Ivory Coast [26], Madagascar [26], Mali [26], Senegal [26], Somalia [17,18,27], Afghanistan [18], Iran [18], Iraq [18], Syria [18], Lebanon [17,18], Asia [27], Pakistan [17,18,21,25,27], India [25], Bangladesh [25], , Philippines [18], Thailand [18], Vietnam [18,27], China [18,26], the Americas [26],

			North America [27], USA [27], South America [27], Caribbean [25], Haiti [26], Oceania [26].
			**Other:** All [16], high-income countries [16], middle income-countries [16], low-income countries [16], OECD [29], other non-OECD [29], EU/EEA [29], White [25]
Under-5<br>(1 study)	Congenital malformations [30], external causes [30], perinatal causes [30], Sudden Infant Death Syndrome [30]	Denmark [30]	**European**: Norway [30], Sweden [30], Former Yugoslavia [30]
			**Non-European:** Turkey [30], Somalia [30], Afghanistan [30], Iraq [30], Iran [30], Lebanon [30], Pakistan [30]
Adult<br>(21 studies)	Accidents & injuries [1,31,32], alcohol-related [32,33], all external causes [31,32,34], all natural causes [34], cancers (all) [1,31,32,35–37], cancer (lung) [31,32,36–38], cancer (other specific sites) [32,37,38], cardiovascular diseases [31,32,38], circulatory diseases [1], coronary heart disease [39], infectious diseases [32,36], other diseases & medical conditions [1], other external causes [1], respiratory diseases [31,32], substance misuse [1,33], suicide [1,31,32,40–43]	Belgium [36,42,44], France [45,46], Netherlands [32,35,47], Norway [40], Sweden [1,34,37,39,41,43],<br>United Kingdom [48]	**European**: Europe (EU) [43], Europe (non-EU) [43], Western Europe [37,40,41], Nordic [1,33,37,40,43], Denmark [37,39], Finland [1,34,37,39,41], Norway [37,39], other Western Europe [39], Austria [37], Germany [37], France [36–38], Netherlands [36–38], Ireland [31], United Kingdom [37], Southern Europe [39,41,45,46], Greece [37], Spain [37], Italy [36–38,42], Central & Eastern Europe [1], Eastern Europe [34,37,40,41], Central Europe [39], Eastern Europe [39], former Yugoslavia [34,37] Baltic states [39], Russia [37,39], Estonia [37], Poland [37], Romania [37]
			**Non-European:** Turkey [35,36,38,39,42], Africa [40], Northern Africa [45,46], Morocco/Tunisia [44], Sub-Saharan Africa [1,36,44], Black Africa [48], the Middle East [1,34,41], Morocco [35,38,42], Asia (inc. Turkey) [40], China & other Asia [48], India [48], Pakistan & Bangladesh [48], Asia [1,37], Indonesia (the Moluccans) [32,47], North America [37], United States [37], North America & Oceania [40], Central & Southern America [1,40], Suriname [35], Antilles/Aruba [35], Black Caribbean [48], non-European [41], other non-European [34]
			**Other:** All [1,35,37,43], Black other [48], other Western [1], Western [34,36], non-Western [36], non-Nordic [33]
