# Supplementary material for: Elevated mortality among the second-generation (children of migrants) in Europe: what is going wrong? A review
Source: Br Med Bull. 2023 Nov 1;148(1):5–21. doi: 10.1093/bmb/ldad027 (PMC10724460; doi:10.1093/bmb/ldad027)
Supplement: Suppl_file_S3_ldad027 [file suppl_file_s3_ldad027.pdf]

Supplementary file S3. Characteristics of the individual EARLY LIFE mortality studies.

Lead author	Year	Country	Data source	Definition of second-generation	Variables used to define second-generation	Age range	Period
Damsted Rasmussen	2021	Denmark	Danish national population registers	Born in Denmark to a foreign-born mother (with two foreign-born parents)	Country of birth; mother's country of birth; mother's mother's country of birth; mother's father's country of birth	22 or more weeks of gestation (stillbirth); 0-364 days of life (infant)	2005-2016
Wallace	2021	France	French Permanent Demographic Sample (EDP) (census and civil register data)	Born in France to at least one parent born abroad	Country of birth; mother's country of birth; father's country of birth	0-364 days of life (infant)	2008-2017
Vik	2020	Norway	Norwegian national population registers	Born in Norway to a foreign-born mother (with two foreign-born parents)	Country of birth; mother's country of birth; mother's mother's country of birth; mother's father's country of birth	At least 22 weeks gestational age OR at least 500g in birthweight if gestational age is missing	1990-2016
Opondo	2020	England & Wales	Statutory birth and death registration data	Born in England & Wales to a foreign-born mother with non-White British ethnicity	Country of birth; mother's country of birth; mother's ethnicity	0-28 days of life (neonatal); 0-364 days of life (infant)	2006-2012
Wanner	2020	Switzerland	Swiss populaton register; Swiss civil register;	Born in Switzerland to a foreign-born mother	Country of birth; mother's country of birth; mother's permit of residence	0-364 days of life (infant)	2011-2017
Vik	2019	Norway	Norwegian national population registers	Born in Norway to a foreign-born mother with two foreign-born parents	Country of birth; mother's country of birth; mother's mother's country of birth; mother's father's country of birth	At least 22 weeks of gestation OR a birthweight of at least 500g (if data on gestational age were missing)	1990-2013
Wanner	2017	Switzerland	Vital statistics (birth and death registers)	Born in Switzerland with a foreign citizenship at birth	Country of birth; child's citizenship at birth	0-27 days of life (neonatal); 0-364 days of life (infant)	1980-2011
Racape	2016	Belgium	Belgian civil registers	Birth in Belgium to a mother with non-Belgian nationality (with mother's who have acquired Belgian nationality differentiated e.g.., Sub-Saharan Africa versus Sub-Saharan Africa naturalised Belgian)	Mother's nationality at her own birth; mother's nationality at the birth of the child	At least 22 weeks gestational age to 7 days old	1998-2010
Barona-Vilar	2014	Spain	Regional perinatal mortality registry of Valencia	Birth in Spain to a foreign-born mother	Country of birth; mother's country of birth	At least 22 weeks gestational age (stillbirth); 0-28 days of life (neonatal)	2005-2008
Gillet	2014	Belgium	Belgian civil registers	Born in Belgium to a mother with a non-Belgian nationality at the mother's own birth	Country of birth; mother's nationality at mother's own birth	22 or more weeks of gestation AND weighing 500g or more (fetal); 0-364 days of life (infant)	2004-2008
Sørbye	2014	Norway	Norwegian national population registers	Born in Norway to a foreign-born mother with two foreign-born parents	Country of birth; mother's country of birth; mother's mother's country of birth; mother's father's country of birth	Death before or during birth (stillbirth); 0-364 days of life (infant)	1995-2010
Kinge	2014	Norway	Norwegian national population registers	Born in Norway to a foreign-born mother	Country of birth; mother's country of birth	0-364 days of life (infant)	1992-2010
Racape	2013	Belgium	Belgian civil registers	Birth in Belgium to a mother with non-Belgian nationality (with mother's who have acquired Belgian nationality differentiated e.g.., Sub-Saharan Africa versus Sub-Saharan Africa naturalised Belgian)	Mother's nationality at her own birth; mother's nationality at the birth of the child	22 or more weeks of gestation to 0-6 days of life (perinatal)	1998-2008
Naimy	2013	Norway	Norwegian national population registers	Born in Norway to a foreign-born mother	Country of birth; mother's country of birth	22 weeks of gestation to 0-6 days of life (perinatal)	1986-2005
Luque-Fernandez	2013	Spain	Vital-statistics database	Born in Spain to a foreign-born mother	Country of birth; mother's country of birth	At least 22 weeks of gestation OR a birthweight of at least 500g	2007-2010
Reeske	2011	Germany	Nationwide perinatal database	Born in Germany to a foreign-born mother	Country of birth; mother's country of birth	Birth without vital signs after delivery AND with a birthweight of at least 500g	2004-2007
Ravelli	2011	The Netherlands	The Netherlands Perinatal Registry	Born in the Netherlands to an ethnic minority mother	Country of birth; mother's race; mother's country of birth	At least 24 weeks of gestation AND at least 500g in birthweight (stillbirth); 0-6 days of life (early neonatal); At least 24 weeks of gestation AND at least 500g in birthweight up to 7 days of life (pernatal)	2000-2006
Ekéus	2011	Sweden	Swedish national population registries	Born in Sweden to a foreign-born mother	Country of birth; mother's country of birth	At least 28 weeks of gestation (stillbirth)	1992-2005
Pedersen	2011	Denmark	Danish national population registers	Born in Denmark to a foreign-born mother	Country of birth; mother's country of birth	0-5 years of life	1973-2004
Racape	2010	Belgium	Belgian civil registers	Birth in Belgium to a mother with non-Belgian nationality	Country of birth; maternal nationality at delivery	22 or more weeks of gestation (fetal); 0-6 days of life (early neonatal); 7-27 days of life (late neonatal); 28--364 days of life (post neonatal)	1998-2006
Villadsen	2010	Austria; Belgium; Denmark; England & Wales; Germany; the Netherlands; Norway; Sweden; Switzerland	National register (Austria); national birth register (Belgium); national medical birth registry (Denmark); national statistics (England & Wales); state register and estimates from previous publication (Germany); estimates from previous publication (the Netherlands); national medical birth registry (Norway); national birth	Born in one of the name countries to a child with foreign nationality (Austria; Germany) OR a mother with a foreign nationality (Switzerland) OR a foreign-born mother (Belgium; Denmark; England & Wales; the Netherlands; Norway; Sweden)	Country of birth; child's nationality at birth (Austria: Germany): mother's nationality at birth of the child (Switzerland); mother's country of birth (Belgium; Denmark; England & Wales; the Netherlands; Norway; Sweden)	At least 500g in birthweight (Austria; Belgium); at least 500g in birthweight after 1 April 1994--at least 1000g in birthweight on and before 1 April 1994 (Germany); at least 22 weeks of gestation (Norway); at least 22 weeks of gestation OR at least 500g in birthweight (Switzerland); at least 24 weeks of gestation (England & Wales; the Netherlands); at least 28 weeks of gestation (Denmark; Sweden)	1995-2000 (Denmark; the Netherlands) 2000-2005 (Austria; Belgium); 1990-2004 (England & Wales); 1990-1997 (Germany); 1990-2003 (Norway); 1992-2005 (Sweden); 1990-2005 (Switzerland)
Villadsen	2009	Denmark	Danish national population registers	Born in Denmark to a foreign-born mother	Country of birth; mother's country of birth	At least 28 weeks of gestation (stillbirth); 0-364 days of life (infant); 0-27 days of life (neonatal); 28-264 days of life (postneonatal)	1981-2003
