# Supplementary material for: Elevated mortality among the second-generation (children of migrants) in Europe: what is going wrong? A review
Source: Br Med Bull. 2023 Nov 1;148(1):5–21. doi: 10.1093/bmb/ldad027 (PMC10724460; doi:10.1093/bmb/ldad027)
Supplement: Suppl_file_S4_ldad027 [file suppl_file_s4_ldad027.pdf]

Supplementary file S4. Characteristics of the individual ADULT mortality studies.

Lead author	Year	Country	Data source	Definition of second-generation	Variables used to define second-generation	Age range	Period
Wallace	2023	Sweden	Swedish national population registers	Born in Sweden to at least one foreign-born parent	Country of birth; mother's country of birth; father's country of birth	16-42	1990-2016
Wallace	2022	Sweden	Swedish national population registers	Born in Sweden to at least one foreign-born parent	Country of birth; mother's country of birth; father's country of birth	15-44	1997-2016
Saarela	2020	Sweden	Swedish & Finnish national population registers	Born in Sweden to two foreign-born parents<br>Born in Sweden to foreign-born father only<br>Born in Sweden to foreign-born mother only<br>Registered Finnish with two registered Swedish parents<br>Registered Finnish with registered Swedish father only<br>Registered Finnish with registered Swedish mother only	Country of birth; mother's country of birth; father's country of birth (Sweden) ; ethnolinguistic registration; mother's ethnolinguistic registration; father's ethnolinguistic registration (Finland)	17+	1971-2017
Lundgren	2019	Sweden	Swedish national population registers	Born in Sweden to at least one foreign-born parent	Country of birth; mother's country of birth; father's country of birth	18+	2003-2017
Khlat	2019	France	Linked census and mortality register data	Born in France to two parents born abroad	Country of birth; mother's country of birth; father's country of birth	18-64	1999-2010
Guillot	2019	France	Linked census and mortality register data	Born in France to two parents born abroad	Country of birth; mother's country of birth; father's country of birth	18-64	1999-2010
Bodewes	2018	The Netherlands	Cause of death registry: municipal population registers	Born in the Netherlands to foreign-born parents (*not specified if one or both*)	Country of birth; mother's country of birth; father's country of birth; mother's surname; father's surname	*NS*	2000-2013
Hemelrijck	2017	Belgium	Linked census and mortality register data	Born in Belgium with "foreign-origins" (as defined by variables in the next column)	Country of birth; current nationality; nationality at birth; parents' nationality at birth	40-69	2001-2011
Puzo	2017	Norway	Norwegian national population registers	Born in Norway to two foreign-born parents	Country of birth; mother's country of birth; father's country of birth	*NS*	1969-2012
Bauwelinck	2017	Belgium	Linked census and mortality register data	Born in Belgium with "foreign-origins" (as defined by variables in the next column)	Country of birth; current nationality; nationality at birth; parents' nationality at birth	18-64	2001-2011
Wallace	2016	England & Wales	Linked census and mortality register data (1% sample)	Ethnic minority (i.e., not White British) born in England & Wales	Country of birth; ethnicity	20+	1991-2012
Manhica	2015	Sweden	Swedish national population registers	Born in Sweden to two foreign-born parents<br>Born abroad to two foreign-born parents having arrived before age 7	Country of birth; mother's country of birth; father's country of birth;<br>age at arrival (if born abroad)	18-65	1990-2008
Vandenheede	2015	Belgium	Linked census and mortality register data	Born in Belgium with "foreign-origins" (as defined by variables in the next column)	Country of birth; current nationality; nationality at birth; parents' nationality at birth	25-54	2001-2011
Di Thiene	2015	Sweden	Swedish national population registers	Born in Sweden with two foreign-born parents<br>Born in Sweden with one foreign-born and one native-born parent	Country of birth; mother's country of birth; father's country of birth	16-50	2005-2010
De Grande	2014	Belgium	Linked census and mortality register data	Born in Belgium with at least one foreign-born parent	Country of birth; mother's country of birth; father's country of birth	15-34	2001-2006
Ho	2007	The Netherlands	Cause of death registry: municipal population registers	Born in the Netherlands to two foreign-born parents	Country of birth; mother's country of birth; father's country of birth	20+	1995-2000
Stirbu	2006	The Netherlands	Cause of death registry: municipal population registers	Born in the Netherlands to at least one foreign-born parent	Country of birth; mother's country of birth; father's country of birth	0-74	1995-2000
Sundquist	2006	Sweden	Swedish national population registers	Born in Sweden with at least one foreign-born parent	Country of birth; mother's country of birth; father's country of birth	25-69	1987-2001
Hemminki	2002	Sweden	Swedish national population registers	Born in Sweden to foreign-born father<br>Born in Sweden to foreign-born mother	Country of birth; mother's country of birth; father's country of birth	0-66	1961-1998
Hjern	2002	Sweden	Swedish national population registers	Born in Sweden with at least one foreign-born parent	Country of birth; mother's country of birth; father's country of birth	10-68	1990-1998
Harding	1996	England & Wales	Linked census and mortality register data (1% sample)	Born in England & Wales to at least one foreign-born parent<br>Born in England & Wales to two foreign-born parents<br>Born in England & Wales to foreign-born father only<br>Born in England & Wales to foreign-born mother only	Country of birth; mother's country of birth; father's country of birth	15+	1971-1989
