# Supplementary material for: Elevated mortality among the second-generation (children of migrants) in Europe: what is going wrong? A review
Source: Br Med Bull. 2023 Nov 1;148(1):5–21. doi: 10.1093/bmb/ldad027 (PMC10724460; doi:10.1093/bmb/ldad027)
Supplement: Supplementary_materials_ldad027 [file supplementary_materials_ldad027.pdf]

Supplementary file S1. Database searches

Scopus

(TITLE-ABS-KEY (mortality OR death OR infant AND mortality OR perinatal AND mortality OR neonatal AND mortality OR infant AND death OR perinatal AND death OR neonatal AND death OR life AND expectancy OR longevity OR surviv* OR stillbirth) AND TITLE-ABS-KEY (second-generation OR child* OR descendant* OR offspring) AND NOT TITLE-ABS-KEY (cell* OR surgery OR molecul* OR clinic* OR antipsy* OR drug* OR treatment OR protein OR kidney OR vaccin* OR pollut*)) AND NOT TITLE-ABS-KEY ("soil" OR "vaccin*" OR "*biolog*" OR "bird*" OR "cancer" OR "veteri*" OR "DNA" OR "genetic*" OR "gene" OR "genes" OR "cell*" OR "tox*" OR "medic*" OR "animal*" OR "disease*") AND NOT (SUBJAREA (agri OR bioc OR immu OR neur OR phar) OR SUBJAREA (ceng OR chem OR comp OR eart OR ener OR engi OR envi OR mate OR math OR phys))

Web of Science

((ALL=(mortality OR death OR perinatal mortality OR neonatal mortality OR infant death OR perinatal death OR neonatal death OR longevity OR surviv* OR stillbirth)) AND ALL=(second-generation OR descendants of immigrants OR descendants of migrants OR children of immigrants OR children of migrants OR offspring of immigrants OR offspring of migrants OR immigrant descendants OR migrant descendants OR immigrant children OR migrant children OR immigrant offspring OR migrant offspring))

PubMed

((mortality OR death OR infant mortality OR perinatal mortality OR neonatal mortality OR infant death OR perinatal death OR neonatal death OR life expectancy OR longevity OR surviv* OR stillbirth) AND (second-generation OR descendants of immigrants OR descendants or migrants OR children of immigrants OR children of migrants OR offspring of immigrants OR offspring of migrants OR immigrant descendants OR migrant descendants OR immigrant children OR migrant children OR immigrant offspring OR migrant offspring) AND ((excludepreprints[Filter]) AND (humans[Filter]) AND (english[Filter])) AND ((excludepreprints[Filter]) AND (humans[Filter]) AND (english[Filter])) AND ((excludepreprints[Filter]) AND (humans[Filter]) AND (english[Filter]))) NOT (randomized) NOT (drug*) NOT (trial) NOT (case report) NOT (clinic) NOT (antipsy*) NOT (surgery) AND ((excludepreprints[Filter]) AND (humans[Filter]) AND (english[Filter])) AND ((excludepreprints[Filter]) AND (humans[Filter]) AND (english[Filter])) AND ((excludepreprints[Filter]) AND (humans[Filter]) AND (english[Filter])) AND ((excludepreprints[Filter]) AND (humans[Filter]) AND (english[Filter])) AND ((excludepreprints[Filter]) AND (humans[Filter]) AND (english[Filter])) AND ((excludepreprints[Filter]) AND (humans[Filter]) AND (english[Filter])) AND ((excludepreprints[Filter]) AND (humans[Filter]) AND (english[Filter])) AND ((excludepreprints[Filter]) AND (humans[Filter]) AND (english[Filter]))

Supplementary file S2. Basic information on the studies incorporated into the review.

**Outcome**	**Cause-of-death**	**Host country**	**Birth country/region of migrant parents**
Perinatal (5 studies; 2 systematic reviews**: 2 meta-analyses**)	Asphyxia and unexpected deaths prior to onset of labour [13], congenital malformations [10,12,13], infections [10], intrapartum events [13], prematurity and foetal growth [10,13]	Spain [10], Belgium [11–13], Norway [14]	**European**: All EU-27 [11], new EU-27 [12], other EU-15 [12], Western Europe [10], Eastern Europe [10–12]
			**Non-European:** Turkey [11–13], Maghreb region [11], Northern Africa [10,12], Morocco [13], Sub-Saharan Africa [10–13], Somalia [14], Afghanistan [14], Iraq [14], Asia [10], Pakistan [14], Philippines [14], Sri Lanka [14], Thailand [14], Vietnam [14], Latin America [10]
Stillbirth (11 studies; 1 systematic review**: 1 meta-analysis**)		Austria [15], Belgium [15,16], Denmark [15,17,18], Germany [15,19], Netherlands [20], Norway [5,15,21,22], Spain [23], Sweden [15,24], Switzerland [15], United Kingdom [15]	**European**: EU [23], Western Europe [24], Germany [18], Iceland [18], Norway [18], Denmark [22], Sweden [18,22], Mediterranean countries [19], Eastern Europe [24], former Yugoslavia [17,18,22], other Europe [23], Poland [18,22], Romania [18], Russia [22], Ukraine [18], Lithuania [18],
			**Non-European:** Turkey [15,17,18,22], Turkey/Morocco [20], Africa [20,24], Morocco [22], Sub-Saharan Africa [23], Somalia [17,18,22], Middle East [24], Afghanistan [18,22], Iran [18,22], Iraq [18,22], Lebanon [17,18], Syria [18] Asia [20,24], East Asia [20], Asia & Oceania [23], Pakistan [17,18,21,22], Philippines [18,22], Sri Lanka [22], Thailand [18,22], Vietnam [18,22], China [18], Latin America [24], South America & the Caribbean [23]
			**Other:** All [5,16], high-income countries [16], middle income-countries [16], low-income countries [16], Europe & America [19], other Western [20], other non-Western [20]
Neonatal (4 studies; 3 systematic reviews**; 1 meta-analysis**)		Belgium [16], Netherlands [20], Spain [10], United Kingdom [25]	**European**: Western Europe [10], other Western [20], Eastern Europe [10]
			**Non-European:** Turkey/Morocco [20], Africa [20], Northern Africa [10], Sub-Saharan Africa [10,25], Asia [10], East Asia [20], South Asia [20], India [25], Pakistan [25], Bangladesh [25], Latin America [10], Caribbean [25]
			**Other:** All [16], high-income countries [16], middle income-countries [16], low-income countries [16], White [25], other non-Western [20],
Infant (9 studies; 2 systematic reviews**; 2 meta-analyses**)	Congenital malformations [17], , perinatal causes [17], Sudden Infant Death Syndrome [17]	Denmark [17,18], Belgium [16] France [26], Norway [21,27], Switzerland [28,29], United Kingdom [25]	**European**: Europe [27], Germany [18,27,28], Iceland [18], Norway [18], Sweden [18,27], Denmark [27], United Kingdom [27], France [28], Italy [28], Portugal [28], Spain [28], Eastern Europe [26], former Yugoslavia [17,18], Kosovo [28], Macedonia [28], Poland [18,26,27], Romania [18,26], Russia [26], Lithuania [18], Ukraine [18]
			**Non-European:** Turkey [17,18,26–28], Africa [27], Northern Africa [26], Algeria [26], Morocco [26], Tunisia [26], Sub-Saharan Africa [25,26], Western Africa [26], Cameroon [26], Comoros [26], Democratic Republic of Congo [26], Guinea [26], Ivory Coast [26], Madagascar [26], Mali [26], Senegal [26], Somalia [17,18,27], Afghanistan [18], Iran [18], Iraq [18], Syria [18], Lebanon [17,18], Asia

			[27], Pakistan [17,18,21,25,27], India [25], Bangladesh [25], , Philippines [18], Thailand [18], Vietnam [18,27], China [18,26], the Americas [26], North America [27], USA [27], South America [27], Caribbean [25], Haiti [26], Oceania [26].
			**Other:** All [16], high-income countries [16], middle income-countries [16], low-income countries [16], OECD [29], other non-OECD [29], EU/EEA [29], White [25]
Under-5<br>(1 study)	Congenital malformations [30], external causes [30], perinatal causes [30], Sudden Infant Death Syndrome [30]	Denmark [30]	**European**: Norway [30], Sweden [30], Former Yugoslavia [30]
			**Non-European:** Turkey [30], Somalia [30], Afghanistan [30], Iraq [30], Iran [30], Lebanon [30], Pakistan [30]
Adult<br>(21 studies)	Accidents & injuries [1,31,32], alcohol-related [32,33], all external causes [31,32,34], all natural causes [34], cancers (all) [1,31,32,35–37], cancer (lung) [31,32,36–38], cancer (other specific sites) [32,37,38], cardiovascular diseases [31,32,38], circulatory diseases [1], coronary heart disease [39], infectious diseases [32,36], other diseases & medical conditions [1], other external causes [1], respiratory diseases [31,32], substance misuse [1,33], suicide [1,31,32,40–43]	Belgium [36,42,44], France [45,46], Netherlands [32,35,47], Norway [40], Sweden [1,34,37,39,41,43], United Kingdom [48]	**European**: Europe (EU) [43], Europe (non-EU) [43], Western Europe [37,40,41], Nordic [1,33,37,40,43], Denmark [37,39], Finland [1,34,37,39,41], Norway [37,39], other Western Europe [39], Austria [37], Germany [37], France [36–38], Netherlands [36–38], Ireland [31], United Kingdom [37], Southern Europe [39,41,45,46], Greece [37], Spain [37], Italy [36–38,42], Central & Eastern Europe [1], Eastern Europe [34,37,40,41], Central Europe [39], Eastern Europe [39], former Yugoslavia [34,37] Baltic states [39], Russia [37,39], Estonia [37], Poland [37], Romania [37]
			**Non-European:** Turkey [35,36,38,39,42], Africa [40], Northern Africa [45,46], Morocco/Tunisia [44], Sub-Saharan Africa [1,36,44], Black Africa [48], the Middle East [1,34,41], Morocco [35,38,42], Asia (inc. Turkey) [40], China & other Asia [48], India [48], Pakistan & Bangladesh [48], Asia [1,37], Indonesia (the Moluccans) [32,47], North America [37], United States [37], North America & Oceania [40], Central & Southern America [1,40], Suriname [35], Antilles/Aruba [35], Black Caribbean [48], non-European [41], other non-European [34]
			**Other:** All [1,35,37,43], Black other [48], other Western [1], Western [34,36], non-Western [36], non-Nordic [33]

Supplementary file S3. Characteristics of the individual EARLY LIFE mortality studies.

Lead author	Year	Country	Data source	Definition of second-generation	Variables used to define second-generation	Age range	Period
Damsted Rasmussen	2021	Denmark	Danish national population registers	Born in Denmark to a foreign-born mother (with two foreign-born parents)	Country of birth; mother's country of birth; mother's mother's country of birth; mother's father's country of birth	22 or more weeks of gestation (stillbirth); 0-364 days of life (infant)	2005-2016
Wallace	2021	France	French Permanent Demographic Sample (EDP) (census and civil register data)	Born in France to at least one parent born abroad	Country of birth; mother's country of birth; father's country of birth	0-364 days of life (infant)	2008-2017
Vik	2020	Norway	Norwegian national population registers	Born in Norway to a foreign-born mother (with two foreign-born parents)	Country of birth; mother's country of birth; mother's mother's country of birth; mother's father's country of birth	At least 22 weeks gestational age OR at least 500g in birthweight if gestational age is missing	1990-2016
Opondo	2020	England & Wales	Statutory birth and death registration data	Born in England & Wales to a foreign-born mother with non-White British ethnicity	Country of birth; mother's country of birth; mother's ethnicity	0-28 days of life (neonatal); 0-364 days of life (infant)	2006-2012
Wanner	2020	Switzerland	Swiss populaton register; Swiss civil register;	Born in Switzerland to a foreign-born mother	Country of birth; mother's country of birth; mother's permit of residence	0-364 days of life (infant)	2011-2017
Vik	2019	Norway	Norwegian national population registers	Born in Norway to a foreign-born mother with two foreign-born parents	Country of birth; mother's country of birth; mother's mother's country of birth; mother's father's country of birth	At least 22 weeks of gestation OR a birthweight of at least 500g (if data on gestational age were missing)	1990-2013
Wanner	2017	Switzerland	Vital statistics (birth and death registers)	Born in Switzerland with a foreign citizenship at birth	Country of birth; child's citizenship at birth	0-27 days of life (neonatal); 0-364 days of life (infant)	1980-2011
Racape	2016	Belgium	Belgian civil registers	Birth in Belgium to a mother with non-Belgian nationality (with mother's who have acquired Belgian nationality differentiated e.g.., Sub-Saharan Africa versus Sub-Saharan Africa naturalised Belgian)	Mother's nationality at her own birth; mother's nationality at the birth of the child	At least 22 weeks gestational age to 7 days old	1998-2010
Barona-Vilar	2014	Spain	Regional perinatal mortality registry of Valencia	Birth in Spain to a foreign-born mother	Country of birth; mother's country of birth	At least 22 weeks gestational age (stillbirth); 0-28 days of life (neonatal)	2005-2008
Gillet	2014	Belgium	Belgian civil registers	Born in Belgium to a mother with a non-Belgian nationality at the mother's own birth	Country of birth; mother's nationality at mother's own birth	22 or more weeks of gestation AND weighing 500g or more (fetal); 0-364 days of life (infant)	2004-2008
Sørbye	2014	Norway	Norwegian national population registers	Born in Norway to a foreign-born mother with two foreign-born parents	Country of birth; mother's country of birth; mother's mother's country of birth; mother's father's country of birth	Death before or during birth (stillbirth); 0-364 days of life (infant)	1995-2010
Kinge	2014	Norway	Norwegian national population registers	Born in Norway to a foreign-born mother	Country of birth; mother's country of birth	0-364 days of life (infant)	1992-2010
Racape	2013	Belgium	Belgian civil registers	Birth in Belgium to a mother with non-Belgian nationality (with mother's who have acquired Belgian nationality differentiated e.g.., Sub-Saharan Africa versus Sub-Saharan Africa naturalised Belgian)	Mother's nationality at her own birth; mother's nationality at the birth of the child	22 or more weeks of gestation to 0-6 days of life (perinatal)	1998-2008
Naimy	2013	Norway	Norwegian national population registers	Born in Norway to a foreign-born mother	Country of birth; mother's country of birth	22 weeks of gestation to 0-6 days of life (perinatal)	1986-2005
Luque-Fernandez	2013	Spain	Vital-statistics database	Born in Spain to a foreign-born mother	Country of birth; mother's country of birth	At least 22 weeks of gestation OR a birthweight of at least 500g	2007-2010
Reeske	2011	Germany	Nationwide perinatal database	Born in Germany to a foreign-born mother	Country of birth; mother's country of birth	Birth without vital signs after delivery AND with a birthweight of at least 500g	2004-2007
Ravelli	2011	The Netherlands	The Netherlands Perinatal Registry	Born in the Netherlands to an ethnic minority mother	Country of birth; mother's race; mother's country of birth	At least 24 weeks of gestation AND at least 500g in birthweight (stillbirth); 0-6 days of life (early neonatal); At least 24 weeks of gestation AND at least 500g in birthweight up to 7 days of life (pernatal)	2000-2006
Ekéus	2011	Sweden	Swedish national population registries	Born in Sweden to a foreign-born mother	Country of birth; mother's country of birth	At least 28 weeks of gestation (stillbirth)	1992-2005
Pedersen	2011	Denmark	Danish national population registers	Born in Denmark to a foreign-born mother	Country of birth; mother's country of birth	0-5 years of life	1973-2004
Racape	2010	Belgium	Belgian civil registers	Birth in Belgium to a mother with non-Belgian nationality	Country of birth; maternal nationality at delivery	22 or more weeks of gestation (fetal); 0-6 days of life (early neonatal); 7-27 days of life (late neonatal); 28--364 days of life (post neonatal)	1998-2006
Villadsen	2010	Austria; Belgium; Denmark; England & Wales; Germany; the Netherlands; Norway; Sweden; Switzerland	National register (Austria); national birth register (Belgium); national medical birth registry (Denmark); national statistics (England & Wales); state register and estimates from previous publication (Germany); estimates from previous publication (the Netherlands); national medical birth registry (Norway); national birth	Born in one of the name countries to a child with foreign nationality (Austria; Germany) OR a mother with a foreign nationality (Switzerland) OR a foreign-born mother (Belgium; Denmark; England & Wales; the Netherlands; Norway; Sweden)	Country of birth; child's nationality at birth (Austria: Germany): mother's nationality at birth of the child (Switzerland); mother's country of birth (Belgium; Denmark; England & Wales; the Netherlands; Norway; Sweden)	At least 500g in birthweight (Austria; Belgium); at least 500g in birthweight after 1 April 1994--at least 1000g in birthweight on and before 1 April 1994 (Germany); at least 22 weeks of gestation (Norway); at least 22 weeks of gestation OR at least 500g in birthweight (Switzerland); at least 24 weeks of gestation (England & Wales; the Netherlands); at least 28 weeks of gestation (Denmark; Sweden)	1995-2000 (Denmark; the Netherlands) 2000-2005 (Austria; Belgium); 1990-2004 (England & Wales); 1990-1997 (Germany); 1990-2003 (Norway); 1992-2005 (Sweden); 1990-2005 (Switzerland)
Villadsen	2009	Denmark	Danish national population registers	Born in Denmark to a foreign-born mother	Country of birth; mother's country of birth	At least 28 weeks of gestation (stillbirth); 0-364 days of life (infant); 0-27 days of life (neonatal); 28-264 days of life (postneonatal)	1981-2003

Supplementary file S4. Characteristics of the individual ADULT mortality studies.

Lead author	Year	Country	Data source	Definition of second-generation	Variables used to define second-generation	Age range	Period
Wallace	2023	Sweden	Swedish national population registers	Born in Sweden to at least one foreign-born parent	Country of birth; mother's country of birth; father's country of birth	16-42	1990-2016
Wallace	2022	Sweden	Swedish national population registers	Born in Sweden to at least one foreign-born parent	Country of birth; mother's country of birth; father's country of birth	15-44	1997-2016
Saarela	2020	Sweden	Swedish & Finnish national population registers	Born in Sweden to two foreign-born parents<br>Born in Sweden to foreign-born father only<br>Born in Sweden to foreign-born mother only<br>Registered Finnish with two registered Swedish parents<br>Registered Finnish with registered Swedish father only<br>Registered Finnish with registered Swedish mother only	Country of birth; mother's country of birth; father's country of birth (Sweden) ; ethnolinguistic registration; mother's ethnolinguistic registration; father's ethnolinguistic registration (Finland)	17+	1971-2017
Lundgren	2019	Sweden	Swedish national population registers	Born in Sweden to at least one foreign-born parent	Country of birth; mother's country of birth; father's country of birth	18+	2003-2017
Khlat	2019	France	Linked census and mortality register data	Born in France to two parents born abroad	Country of birth; mother's country of birth; father's country of birth	18-64	1999-2010
Guillot	2019	France	Linked census and mortality register data	Born in France to two parents born abroad	Country of birth; mother's country of birth; father's country of birth	18-64	1999-2010
Bodewes	2018	The Netherlands	Cause of death registry: municipal population registers	Born in the Netherlands to foreign-born parents (*not specified if one or both*)	Country of birth; mother's country of birth; father's country of birth; mother's surname; father's surname	*NS*	2000-2013
Hemelrijck	2017	Belgium	Linked census and mortality register data	Born in Belgium with "foreign-origins" (as defined by variables in the next column)	Country of birth; current nationality; nationality at birth; parents' nationality at birth	40-69	2001-2011
Puzo	2017	Norway	Norwegian national population registers	Born in Norway to two foreign-born parents	Country of birth; mother's country of birth; father's country of birth	*NS*	1969-2012
Bauwelinck	2017	Belgium	Linked census and mortality register data	Born in Belgium with "foreign-origins" (as defined by variables in the next column)	Country of birth; current nationality; nationality at birth; parents' nationality at birth	18-64	2001-2011
Wallace	2016	England & Wales	Linked census and mortality register data (1% sample)	Ethnic minority (i.e., not White British) born in England & Wales	Country of birth; ethnicity	20+	1991-2012
Manhica	2015	Sweden	Swedish national population registers	Born in Sweden to two foreign-born parents<br>Born abroad to two foreign-born parents having arrived before age 7	Country of birth; mother's country of birth; father's country of birth;<br>age at arrival (if born abroad)	18-65	1990-2008
Vandenheede	2015	Belgium	Linked census and mortality register data	Born in Belgium with "foreign-origins" (as defined by variables in the next column)	Country of birth; current nationality; nationality at birth; parents' nationality at birth	25-54	2001-2011
Di Thiene	2015	Sweden	Swedish national population registers	Born in Sweden with two foreign-born parents<br>Born in Sweden with one foreign-born and one native-born parent	Country of birth; mother's country of birth; father's country of birth	16-50	2005-2010
De Grande	2014	Belgium	Linked census and mortality register data	Born in Belgium with at least one foreign-born parent	Country of birth; mother's country of birth; father's country of birth	15-34	2001-2006
Ho	2007	The Netherlands	Cause of death registry: municipal population registers	Born in the Netherlands to two foreign-born parents	Country of birth; mother's country of birth; father's country of birth	20+	1995-2000
Stirbu	2006	The Netherlands	Cause of death registry: municipal population registers	Born in the Netherlands to at least one foreign-born parent	Country of birth; mother's country of birth; father's country of birth	0-74	1995-2000
Sundquist	2006	Sweden	Swedish national population registers	Born in Sweden with at least one foreign-born parent	Country of birth; mother's country of birth; father's country of birth	25-69	1987-2001
Hemminki	2002	Sweden	Swedish national population registers	Born in Sweden to foreign-born father<br>Born in Sweden to foreign-born mother	Country of birth; mother's country of birth; father's country of birth	0-66	1961-1998
Hjern	2002	Sweden	Swedish national population registers	Born in Sweden with at least one foreign-born parent	Country of birth; mother's country of birth; father's country of birth	10-68	1990-1998
Harding	1996	England & Wales	Linked census and mortality register data (1% sample)	Born in England & Wales to at least one foreign-born parent<br>Born in England & Wales to two foreign-born parents<br>Born in England & Wales to foreign-born father only<br>Born in England & Wales to foreign-born mother only	Country of birth; mother's country of birth; father's country of birth	15+	1971-1989
